# Mechanisms and Regulation of DNA-Protein Crosslink Repair During DNA Replication by SPRTN Protease

**DOI:** 10.3389/fmolb.2022.916697

**Published:** 2022-06-15

**Authors:** Megan Perry, Gargi Ghosal

**Affiliations:** ^1^ Department of Genetics, Cell Biology and Anatomy, University of Nebraska Medical Center, Omaha, NE, United States; ^2^ Fred and Pamela Buffett Cancer Center, Omaha, NE, United States

**Keywords:** DPC, DPC proteolysis, Ruijs-Aalfs syndrome, SPRTN protease, translesion synthesis (TLS)

## Abstract

DNA-protein crosslinks (DPCs) are deleterious DNA lesions that occur when proteins are covalently crosslinked to the DNA by the action of variety of agents like reactive oxygen species, aldehydes and metabolites, radiation, and chemotherapeutic drugs. Unrepaired DPCs are blockades to all DNA metabolic processes. Specifically, during DNA replication, replication forks stall at DPCs and are vulnerable to fork collapse, causing DNA breakage leading to genome instability and cancer. Replication-coupled DPC repair involves DPC degradation by proteases such as SPRTN or the proteasome and the subsequent removal of DNA-peptide adducts by nucleases and canonical DNA repair pathways. SPRTN is a DNA-dependent metalloprotease that cleaves DPC substrates in a sequence-independent manner and is also required for translesion DNA synthesis following DPC degradation. Biallelic mutations in SPRTN cause Ruijs-Aalfs (RJALS) syndrome, characterized by hepatocellular carcinoma and segmental progeria, indicating the critical role for SPRTN and DPC repair pathway in genome maintenance. In this review, we will discuss the mechanism of replication-coupled DPC repair, regulation of SPRTN function and its implications in human disease and cancer.

## Introduction

Living organisms require that their genomic sequence be preserved and transferred from one cell to the next to promote survival at the cellular, organism, and species level, a process known as maintenance of genome stability. However, DNA is susceptible to damage by endogenous stressors including DNA replication errors, reactive metabolites and oxygen species and exposure to exogenous agents such as chemotherapeutic agents, radiation, or other environmental toxins. Types of DNA damage include individual base damages such as base mismatches, deamination, apurinic/apyrimidinic (AP) sites, and aberrant methylation or oxidation. DNA damage can also result in single-strand DNA breaks (SSBs), double-strand DNA breaks (DSBs), inter- and intra-strand DNA crosslinks, and bulky DNA-protein crosslink (DPC) adducts (Chatterjee and Walker, 2017). Failure to repair DNA damage can result in genome instability, characterized by the acquisition of DNA mutations and chromosomal breaks and rearrangements that can be deleterious to cells. Genome instability in humans is often associated with pathologies linked to premature aging, cancer predisposition and inherited disorders (Aguilera and Gómez-González, 2008).

The different types of DNA damage require multiple cellular pathways for sensing, removing, and repairing the damage, a process collectively referred to as the DNA damage response. Cells use distinct DNA repair pathways such as mismatch repair, base excision repair (BER), nucleotide excision repair (NER), DNA-protein crosslink repair, single stranded break repair (SSBR), non-homologous end joining (NHEJ), and homologous recombination (HR) to repair damaged DNA. Choice of the DNA repair pathway depends on the type of DNA damage and depending on the complexity of the DNA lesion, repair may involve the coordinated activity of one or more DNA repair pathways (Sancar et al., 2004). Herein, we will review DPC repair with the focus on DPC proteolysis and removal by SPRTN during DNA replication.

## DNA-Protein Crosslinks

DNA needs to be dynamically manipulated by proteins that unwind, nick, read, replicate, and organize the genome for gene expression, replication, and recombination. The Cell Atlas reports that 34% of all human protein-coding genes encode proteins that localize to the nucleoplasm (Thul et al., 2017). Thus, DNA-binding proteins as well as nuclear proteins that do not bind directly to DNA are in close proximity to DNA at all times and are therefore susceptible to trapping by reactive endogenous and exogenous stressors. Covalent crosslinking of proteins to DNA upon damage or at DNA breaks causes the formation of highly variable, bulky lesions known as DNA-protein crosslinks. DPCs are generated by the action of oxygen free radicals, reactive nitrogen species, and reactive aldehydes generated as by-products of cellular respiration and metabolism or by exposure to exogenous DNA damaging agents like ultraviolet (UV) radiation, ionizing radiation (IR), and chemotherapeutic drugs (Tretyakova et al., 2015; Vaz et al., 2017; Fielden et al., 2018).

DPCs are diverse lesions and can be classified on the basis of the crosslinked protein and the chemistry of the crosslink. Here, we provide a broad overview on DPC classification and direct the readers to references (Stingele and Jentsch, 2015; Fielden et al., 2018; Sun et al., 2020a; Wei et al., 2021; Weickert and Stingele, 2022) for more detailed DPC classification. Based on the identity of the crosslinked protein, DPCs are broadly classified into two groups: enzymatic and non-enzymatic. Enzymatic DPCs arise when a DNA-binding enzyme becomes covalently trapped as a reaction intermediate due to the actions of specific chemotherapies or abortive DNA repair processes. Some of the proteins that can become enzymatic DPCs include TOP1 (topoisomerase I), TOP2 (topoisomerase II), DNA polymerase, PARP1 (poly [ADP-ribose] polymerase 1), SPO11 (meiotic recombination protein sporulation 11), HMCES (5-Hydroxymethylcytosine binding, ES-cell-specific), and DNMT1 (DNA methyltransferase) (Santi et al., 1984; Tewey et al., 1984; Hsiang et al., 1985; Hsiang et al., 1989; Jüttermann et al., 1994; Pommier and Marchand, 2011; Mohni et al., 2019). Non-enzymatic DPCs, on the other hand, are formed by the covalent crosslinking of any protein in the vicinity of DNA at the time of exposure to a DPC-inducing agent (Stingele et al., 2017).

**FIGURE 1:**
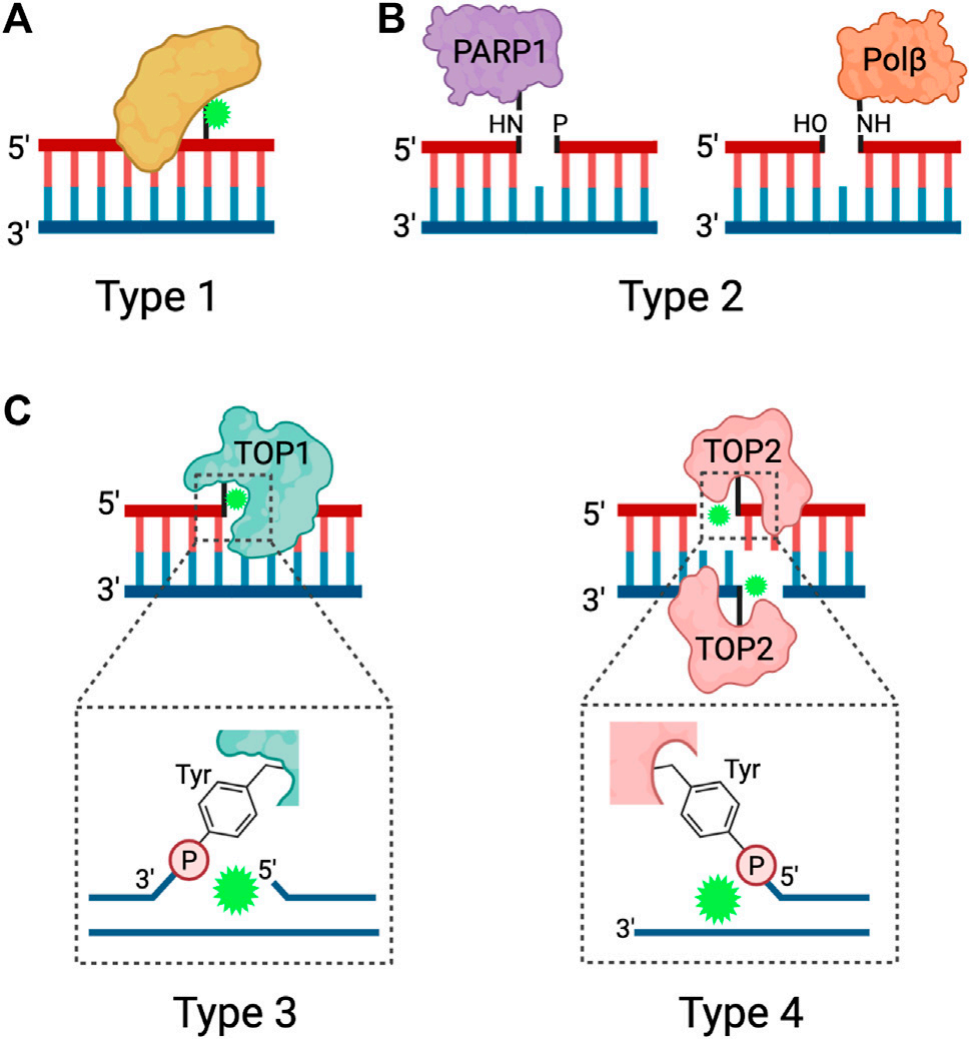
Types of DPCs. Schematic depicting the four types of DPCs. **(A)** Type 1 DPCs are proteins crosslinked to unperturbed duplex DNA, generally induced by nonspecific agents. **(B)** Type 2 DPCs are crosslinked to the ends of SSBs and arise from abortive DNA repair processes. **(C)** Types 3 and 4 DPCs are abortive DNA-topoisomerase DPCs, where TOP1 is crosslinked to the 3ʹ end of a SSB or TOP2 is crosslinked to the 5ʹ ends of a DSB via a phosphotyrosyl bond. Adapted from Nakano et al. (2017), Stingele et al. (2017). Created in BioRender.com.

DPCs can be further classified by the chemistry of the crosslink and subcategorized based on the structure of the DNA flanking the linkage. Type 1 DPCs are formed by covalent crosslinking of proteins to the unperturbed DNA duplex, and are generated by formaldehyde, IR, UV, and platinum-based chemotherapeutic drugs (Figure 1A). Type 1 DPCs represent the most diverse group of DPCs as there is substantial overlap with the broad group of non-enzymatic DPCs. Cellular exposure to IR generates oxygen free radicals that react with proteins and DNA to form DPCs, especially under hypoxic conditions (Stingele and Jentsch, 2015; Nakano et al., 2017). Formaldehyde forms DPCs by reacting with the nucleophilic side chains of lysine and cysteine amino acid (aa) residues to form a reactive Schiff base intermediate that crosslinks with a DNA base, most often guanine (Lu et al., 2010). In addition to environmental and occupational exposures, formaldehyde is produced as a byproduct of histone demethylation (Shi et al., 2004; Tsukada et al., 2006) and AlkB-mediated repair of DNA-methylation damage (Trewick et al., 2002; Shen et al., 2014). Despite the presence of aldehyde dehydrogenases, which can convert formaldehyde to formic acid, the formaldehyde concentration in human blood ranges from 2–3 mg/L (Zhang et al., 2009) and endogenous formaldehyde-induced DPCs are ubiquitously detected in cells (Swenberg et al., 2011).

Trapped DNA Polβ (polymerase beta) and PARP1 at the 5′ and 3′ ends of SSBs, respectively, represent type 2 DPCs (Figure 1B). Polβ and PARP1 participate in the BER pathway downstream of APE1, an endonuclease that generates AP sites. When the burden of AP sites is particularly high or when cells are treated with PARP inhibitors, PARP1 becomes covalently bound to the 3ʹ end of the nicked DNA at the AP site and forms a DPC (Prasad et al., 2019). Polβ functions during short-patch BER but can become trapped to the 5′ end of the SSB when there is oxidative damage that requires activation of long-patch BER (Quiñones et al., 2015).

Type 3 and type 4 DPCs arise from abortive topoisomerase-DNA enzymatic reactions resulting in the crosslinking of TOP1 to the 3′ end of a SSB or TOP2 to the two 5′ ends of a DSB (Ide et al., 2018; Sun et al., 2020a) (Figure 1C). TOP1 resolves DNA supercoiling by nicking DNA and transiently binding to the 3′ end of the SSB, thus allowing the free 5ʹ hydroxyl end to rotate around the intact DNA strand. TOP2 resolves DNA supercoiling, DNA catenanes, and DNA knots by acting as a homodimer to generate a DSB and bind to the 5ʹ end of each strand. The DSB allows for an intact DNA duplex to pass through. When the torsional stress is relieved, TOP1 and TOP2 re-ligate the breaks and are released from the DNA (Stingele and Jentsch, 2015; Pommier et al., 2016). Because the re-ligation of topoisomerase-induced DNA breaks is dependent on precise strand alignment within the DNA-enzyme complex, the reaction is prone to inhibition by structural DNA alterations or treatment with anti-cancer drugs. DNA distortions such as AP sites, intra-strand crosslinks, DNA mismatches and DNA breaks can prevent strand alignment and re-ligation (Sun et al., 2020a). Chemotherapeutic agents known as topoisomerase poisons act as interfacial inhibitors, where a single molecule of the drug is bound at the DNA-enzyme interface to prevent DNA ligation (Pommier and Marchand, 2011). Specifically, camptothecin (CPT) and its derivatives are TOP1 poisons that intercalate at the site of DNA cleavage, and displace the downstream DNA bases to prevent re-ligation, and the TOP2 poisons etoposide (VP16) and teniposide trap TOP2 by binding to the enzyme to prevent re-ligation (Hertzberg et al., 2002; Staker et al., 2002; Wilstermann et al., 2007; Bender et al., 2008).

## Consequences of DPCs

Irrespective of the type or source of the DPC lesion, all DPCs disrupt DNA processes, including transcription and replication (Nakano et al., 2012; Olmedo-Pelayo et al., 2020). DNA epigenetic marks such as 5-formylcytosine bases (5fC) readily forms reversible Schiff base crosslinks with histones or other nuclear proteins *in vitro* and *in vivo*. 5fC-mediated DPCs with histone H2A was shown to block *in vitro* transcription by T7 RNA polymerase (Ji et al., 2019). Induction of topoisomerase-covalent crosslinks (TOP-ccs) and methyltransferase DPCs in *Escherichia coli* caused replication fork stalling (Hong and Kreuzer, 2000; Pohlhaus and Kreuzer, 2005; Kuo et al., 2007). Several studies show that TLS polymerases are stalled by DPCs *in vitro* (Chválová et al., 2007; Nakano et al., 2013; Yeo et al., 2014; Yudkina et al., 2022). TLS polymerases have more flexible active sites than canonical replicative polymerases, allowing for nascent DNA synthesis opposite a DNA lesion and subsequent bypass of the lesion by the replication fork (Sale et al., 2012). Analysis of *in vitro* DNA unwinding by a subcomplex of the replicative helicase CMG (Cdc45/MCM2-7/GINS) comprised of MCM4, MCM6, and MCM7 purified from inset cells revealed that the presence of a DPC ranging from 5–14.1 kDa on the translocating strand stalled helicase progression and DPC lesion bypass by translesion DNA synthesis (TLS) polymerase (Nakano et al., 2013). Subsequently, intricate analysis of replisome dynamics at a DPC in *Xenopus* extracts revealed that when a replication fork encounters a DPC, CMG can slowly bypass the lesion due to accessary helicase activity by RTEL1 (Sparks et al., 2019). However, due to the bulky nature of DPCs, DNA polymerases cannot synthesize DNA past the lesion. CMG slows dramatically after bypass due to helicase-polymerase uncoupling, where CMG continues to translocate and unwind duplex DNA but the polymerase stalls at the DPC. The DPC is then proteolyzed into a DNA-peptide adduct. Therefore, the DPC must be proteolytically degraded to facilitate the bypass or hydrolysis of the DNA-peptide crosslink to resume DNA synthesis (Larsen et al., 2019; Sparks et al., 2019). Unrepaired DPCs or mis-regulation of DPC repair processes generates ssDNA in the vicinity of the DPC and leaves the fork vulnerable to DNA breakage and collapse leading to genome instability, evidenced by stalled forks, micronuclei, sister chromatid exchange, mutagenesis, and gross chromosomal rearrangements, resulting in tumorigenesis and genetic diseases (Oleinick et al., 1987; Titenko-Holland et al., 1996; Kumari et al., 2012; Stingele et al., 2014; Stingele et al., 2017).

## Mechanisms of DPC Repair

### Replication-Dependent DPC Proteolysis is Mediated by DPC Proteases and the Proteasome

**FIGURE 2:**
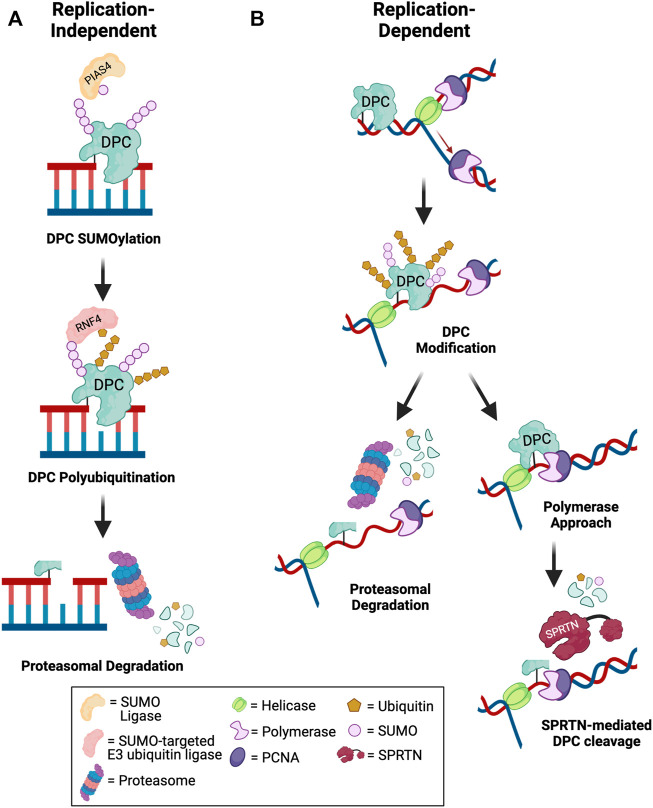
Mechanisms of DPC degradation. Schematic depicting pathways for debulking of DPCs in **(A)** replication-independent and **(B)** replication-dependent contexts. **(A)** Outside of DNA replication, DPCs are targeted for SUMOylation by an E3 SUMO ligase such as PIAS4. Subsequent DPC recognition and polyubiquitination by a SUMO-targeted E3 ubiquitin ligase such as RNF4 promotes proteasomal degradation of the DPC to allow for downstream repair of DNA breaks (Sun et al., 2020c; Liu et al., 2021). **(B)** During DNA replication, fork collision with a DPC triggers helicase-polymerase uncoupling by CMG helicase bypass of the lesion. The DPC may be targeted for modification by ubiquitin or SUMO. Polyubiquitinated DPCs can be targeted for proteasomal degradation. Alternatively, if the polymerase extends the nascent DNA to within a few nucleotides of the lesion, SPRTN-mediated DPC proteolysis is activated to degrade modified or unmodified DPCs. The remaining peptide-DNA adduct can be bypassed by TLS polymerases and repaired post-replication (Larsen et al., 2019; Sparks et al., 2019; Ruggiano et al., 2021). Created in BioRender.com.

Given the complexity of the DPC lesion, which is comprised of the DNA, protein, and covalent crosslink, DPC repair involves a coordinated action of multiple DNA repair pathways. The key steps in DPC repair include DPC proteolysis to generate DNA-peptide crosslinks or adducts, bypass or hydrolysis of DNA-peptide crosslinks followed by repair of the resulting single- or double-stranded DNA breaks. To date, two differentially activated replication-dependent pathways for DPC degradation have been identified: DPC protease and the proteasome (Figure 2).

Wss1 in yeast was the first DPC protease identified that cleaves both enzymatic TOP1-ccs and non-enzymatic formaldehyde-induced DPCs during S-phase (Stingele et al., 2014). Subsequently, SPRTN protease (protein with SprT-like domain at the N-terminus), a structurally similar protein to yeast Wss1, was shown to repair DPCs during replication in mammalian cells (Lopez-Mosqueda et al., 2016; Stingele et al., 2016; Vaz et al., 2016). Studies in *Xenopus* egg extracts showed that both SPRTN and the proteasome can repair replication-coupled DPCs (Figure 2B). Only tandem SPRTN depletion and proteasome inhibition significantly prevented DPC repair, and these two pathways were shown to be activated by distinct mechanisms. In proteasome-mediated DPC degradation, polyubiquitination of the DPC is required for lesion recognition and subsequent degradation, whereas SPRTN-mediated degradation is activated by polymerase stalling within a few nucleotides of the lesion on either the leading or lagging strand, regardless of whether the DPC is polyubiquitinated or not (Larsen et al., 2019). A recent study demonstrated that both DPC ubiquitination and SUMOylation promote DPC repair by SPRTN. DPC ubiquitination signals SPRTN recruitment to DPC sites and DPC SUMOylation prevents activation of HR to promote DPC repair by SPRTN (Ruggiano et al., 2021). Thus, SPRTN promotes repair of unmodified, ubiquitinated, and SUMOylated DPCs. Interestingly, evidence suggests that proteasome and SPRTN-mediated DPC degradation are non-redundant pathways, as depletion of SPRTN also impairs TLS, even if the proteasome is not impaired (Larsen et al., 2019). The active site of the proteasome is buried inside the 20S core particle, and proteolytic cleavage requires that the protein be threaded through the cylindrical particle (Finley, 2009). It is likely that the crosslinked DNA would block full processing of the DPC by the proteasome, thus resulting in a larger DNA-peptide adduct than in the case of SPRTN-mediated proteolysis, as the active site of SPRTN is more solvent exposed and could gain closer access to the crosslinked residues (Li et al., 2019). Therefore, it has been postulated that SPRTN could be required for a second proteolytic cleavage event to promote TLS bypass of DNA-peptide crosslinks, even if the initial proteolysis occurred via the proteasome, which is discussed in a later section of this review (Larsen et al., 2019). Additionally, because of the diverse nature of DPCs, especially those induced by non-specific crosslinkers such as reactive aldehydes, UV and IR, it is possible that not all DPCs will be able to be polyubiquitinated for repair by the proteasome, either due to a lack of lysine residues or because the lysine residue may be buried within the DPC and inaccessible for ubiquitin conjugation.

Although SPRTN is the key DPC protease in S phase, recent studies have identified other proteases that act on specific types of DPCs. FAM111A, a trypsin-like protease domain containing protein was shown to prevent fork stalling and mediate DPC repair. FAM111A interacts with PCNA, exhibits chymotrypsin like protease activity and undergoes DNA-dependent autocleavage in *trans*. FAM111A repairs TOP1-cc and PARP1-DNA crosslinks in mammalian cells (Kojima et al., 2020; Saha et al., 2021). Unlike SPRTN depleted cells, FAM111A knockout cells displayed mild sensitivity to VP16 and formaldehyde treatment suggesting that FAM111A participates in the repair of a subset of DPCs (Kojima et al., 2020). The functional cooperation of SPRTN and FAM111A, if any, in TOP1-cc and PARP1-DNA crosslink proteolysis remains to be examined. Yeast Ddi1 protease was identified in a genetic screen of the tdp1 wss1 mutant that is defective in Top1cc processing. Ddi1 is recruited to Top1cc-like DPC lesion in an S phase-dependent manner and loss of Ddi1 or its putative protease activity hypersensitizes cells to DPC trapping agents independently from Wss1 and 26S proteasome (Serbyn et al., 2020).

## SPRTN Protein Characterization and Function

**FIGURE 3:**
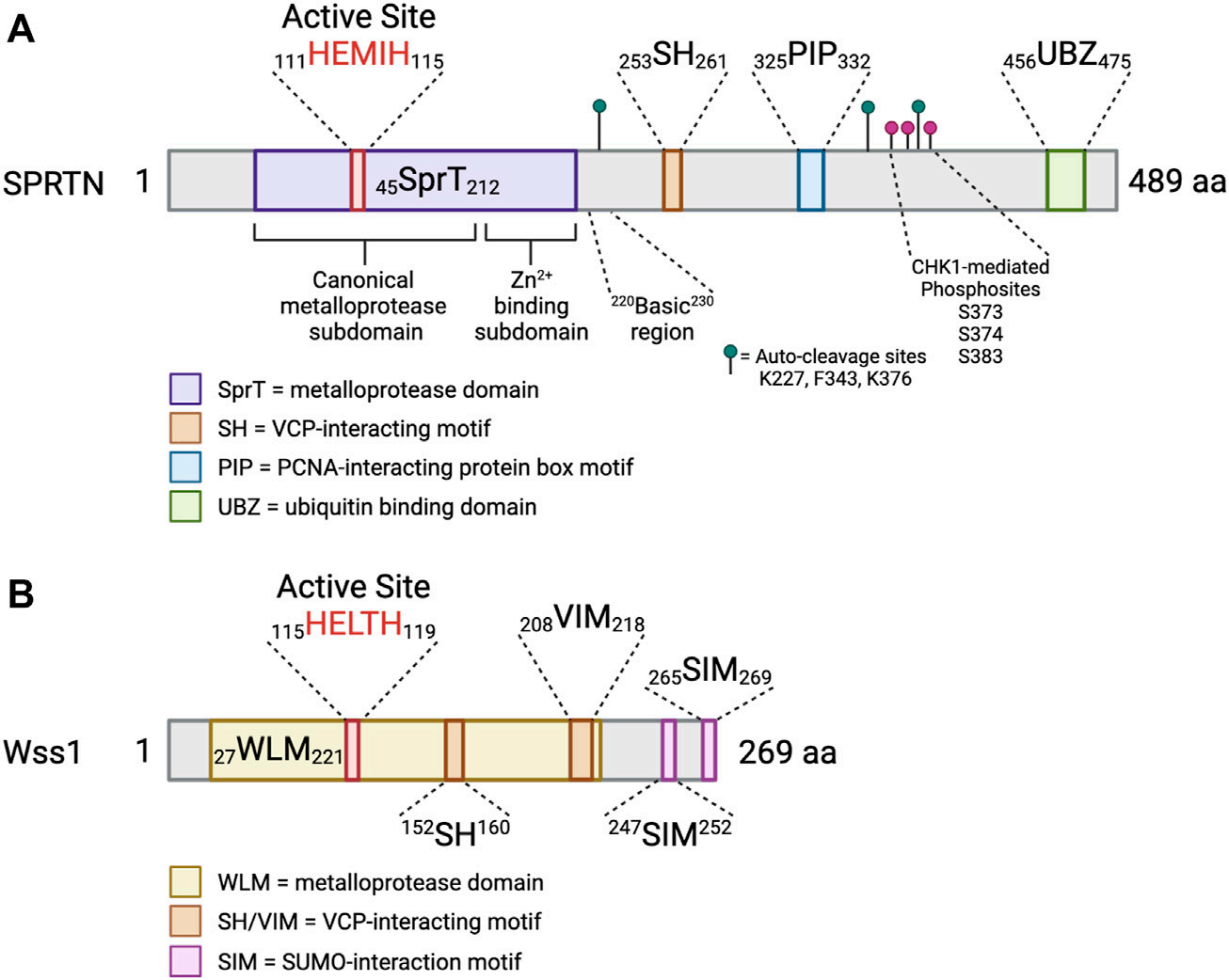
SPRTN is a multidomain protein. Schematic depicting the domains of SPRTN and Wss1 metalloproteases. Small numbers represent aa ranges of indicated domains. **(A)** SprT = metalloprotease domain; SH = VCP-interacting motif; PIP = PCNA-interacting protein box motif; UBZ = ubiquitin binding domain. The SprT domain can be subdivided into the canonical metalloprotease domain (aa 45–166), which contains the conserved HEXXH active site, and the Zn^2+^-binding subdomain which assists in substrate cleavage and has secondary DNA binding function (aa 167–212) (Li et al., 2019). The basic region (aa 220–230) is a critical DNA binding region (Toth et al., 2017; Li et al., 2019). Identified SPRTN auto-cleavage sites are indicated with green lollipops (Vaz et al., 2016), and identified phosphosites are indicated with magenta lollipops (Halder et al., 2019). **(B)** WLM = metalloprotease domain; SH/VIM = VCP-interacting motif; SIM = SUMO-interaction motif (Vaz et al., 2017). Created in BioRender.com.

**FIGURE 4:**
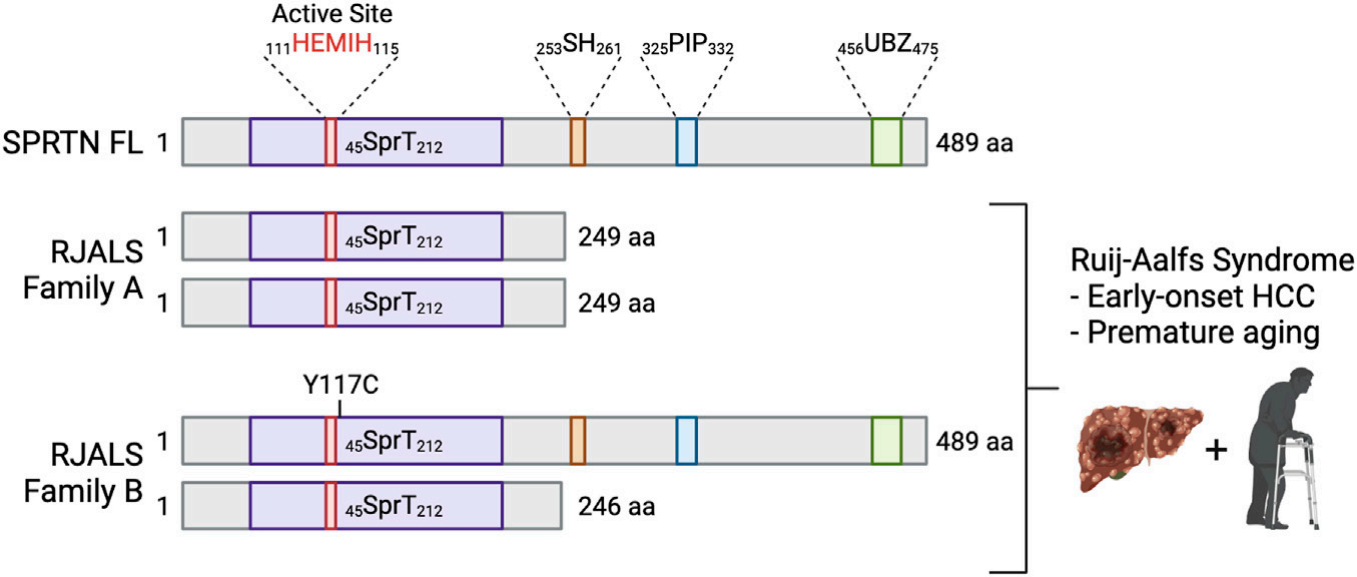
Mutations in *SPRTN* cause Ruijs-Aalfs syndrome. Schematic depicting SPRTN protein products resulting from biallelic mutations identified in RJALS patients. Family A mutants resulted in a premature STOP codon and SPRTN truncation at aa 249 (SPRTN ΔC). Family B compound heterozygous mutations resulted in aa substitution at tyrosine 117 mutated to cysteine (SPRTN Y117C), and a premature STOP codon and truncation at aa 246. RJALS causes early-onset hepatocellular carcinoma and premature aging (Lessel et al., 2014). Created in BioRender.com.

SPRTN (also known as DVC1 and C1orf124) is a 489 aa protein (Figure 3) that has an N-terminal SprT-like domain (aa 45–212) that harbors zinc metalloprotease activity via a conserved ^111^HEXXH^115^ motif, an SH motif (aa 253–261) that mediates interaction with VCP, a PIP box (aa 325–332) mediating interaction with PCNA, and a RAD18-like UBZ domain (aa 456–475) that binds ubiquitin (Centore et al., 2012; Davis et al., 2012; Ghosal et al., 2012; Juhasz et al., 2012; Machida et al., 2012; Mosbech et al., 2012). The UBZ domain is conserved across multiple species, whereas the PIP box is less conserved in lower vertebrates (Centore et al., 2012; Machida et al., 2012) (Figure 3A). The SprT domain harbors RJALS patient SPRTN mutants Y117C and ΔC (1–249 aa) (Figure 4), described in next section (Lessel et al., 2014). Interestingly, the Wss1 metalloprotease in yeast shares similar domain organization to SPRTN. Wss1 has an N-terminal zinc metalloprotease domain, two motifs mediating interaction with Cdc48 (the yeast homolog of VCP), and two C-terminal SUMO-interacting motifs (Figure 3B). Despite the domain structure similarities that resulted in the initial interpretation that *SPRTN* and *Wss1* originated from a common ancestral gene (Stingele et al., 2014; Stingele et al., 2015), there is very little sequence homology between the two proteins. Extended phylogenetic analyses concluded that SPRTN and Wss1 are not the product of divergent evolution, but instead are two distinct proteases whose similarities are a result of convergent evolution (Vaz et al., 2017; Reinking et al., 2020a).

The crystal structure of the human SPRTN SprT domain (aa 26–214) bound to a five nucleotide stretch of ssDNA revealed two subdomains of the SprT domain (Figure 3A). First, the canonical metalloprotease domain (aa 26–166), and second, the Zn^2+^-binding subdomain (ZBD; aa 167–214), wherein each subdomains bind one zinc ion (Vaz et al., 2016; Li et al., 2019). The RJALS mutant Y117C is predicted to disrupt hydrophobic interactions in the ^111^HEXXH^115^ catalytic binding surface, resulting in reduced protease activity (Stingele et al., 2016; Li et al., 2019). The structures of the SPRTN and Wss1 protease domains are largely similar with Wss1 lacking the ZBD subdomain (Stingele et al., 2016; Li et al., 2019).

SPRTN was initially identified in 2012 as a PCNA interacting protein that participates in UV DNA damage repair and regulates bypass of DNA lesions by TLS (Centore et al., 2012; Davis et al., 2012; Ghosal et al., 2012; Juhasz et al., 2012). Wss1 was shown to be a DNA-dependent DPC metalloprotease that repairs Top1-ccs and formaldehyde-induced DPCs by direct cleavage mediated by the metalloprotease domain active site along with partial dependence on Cdc48 interaction. Wss1 also undergoes auto-cleavage (Stingele et al., 2014). Similarly, SPRTN knockdown sensitized cells to treatment with formaldehyde, CPT and VP16 DPC-inducing agents (Lopez-Mosqueda et al., 2016; Stingele et al., 2016; Vaz et al., 2016; Maskey et al., 2017). *Sprtn* knockout (*Sprtn* ^−/−^) MEFs and *C. elegans* mutant strains lacking functional SPRTN (*dvc-1*) were hypersensitive to treatment with DPC inducing agents indicating a role of SPRTN in DPC repair (Lopez-Mosqueda et al., 2016). SPRTN ΔC can partially rescue formaldehyde and CPT sensitivity, but the E112Q or Y117C mutants cannot (Stingele et al., 2016; Vaz et al., 2016). *Sprtn* ^−/−^ in mice results in embryonic lethality. *Sprtn* ^−/−^ MEFs and SPRTN depleted cells displayed increased levels of γH2AX, 53BP1 and RAD51 foci, increased CHK2 activation, and cell proliferation defects with chromatin bridges and micronuclei, indicative of unresolved replication intermediates and unrepaired DNA breaks. This proliferation defect was not rescued by SPRTN E112A (mutation of the catalytic active glutamine to alanine) mutant (Lessel et al., 2014; Maskey et al., 2014). Collectively, these studies demonstrated that SPRTN is essential for embryonic development, DPC repair, faithful DNA replication and maintenance of genome stability.

The requirement of the ^111^HEXXH^115^ metalloprotease motif for complete rescue of replication defects indicated that SPRTN may perform essential proteolytic activity during DNA replication that had yet to be characterized. Similar to Wss1, SPRTN protein displayed auto-proteolysis and substrate cleavage activity, dependent on the presence of DNA in cells and *in vitro* (Stingele et al., 2014; Lopez-Mosqueda et al., 2016; Stingele et al., 2016; Vaz et al., 2016; Li et al., 2019). Auto-cleavage was inhibited by 1,10-phenanthroline, a known inhibitor of Zn^2+^-dependent metalloproteases (Lopez-Mosqueda et al., 2016; Vaz et al., 2016). Auto-cleavage was shown to occur *in trans* and was active in SPRTN FL and SPRTN ΔC, but not SPRTN E112Q or Y117C mutants (Stingele et al., 2016; Vaz et al., 2016; Li et al., 2019). SPRTN also cleaves DNA binding proteins in the presence of DNA *in vitro*. Identified substrates include Histone H1, Histone H2A, Histone H2B, Histone H3, Histone H4, TOP1, TOP2α, and PARP1 crosslinked to DNA (Stingele et al., 2016; Vaz et al., 2016; Li et al., 2019; Saha et al., 2021). SPRTN also cleaved FAN1, HLTF, and yeast RAD5 *in vitro*, but not PCNA, BSA, or RFC (Mórocz et al., 2017). Both SPRTN and Wss1 protease domain structures lack a distinct binding pocket that houses the catalytic center of the ^111^HEXXH^115^ active site, providing evidence for the substrate non-specificity (Stingele et al., 2016; Li et al., 2019).

Analysis of the cleavage sites of SPRTN substrates revealed no common sequence motif, but instead showed that substrates are cleaved in unstructured, positively charged regions that are rich in arginine, lysine, and serine residues. SPRTN auto-cleavage sites are in similar unstructured regions of the SPRTN C-terminus. Mass spectrometry identified SPRTN auto-cleavage sites at residues K227, F343, and K376, although at least five different products are formed (Vaz et al., 2016). The ZBD domain in SPRTN shields the active site and restricts substrate access supporting the observation that SPRTN auto-cleavage and substrate cleavage occurs in flexible, disordered regions of proteins, and suggests that SPRTN substrates may be remodeled prior to cleavage (Stingele et al., 2016; Li et al., 2019).

SPRTN is a replication-coupled DPC metalloprotease (Lopez-Mosqueda et al., 2016; Stingele et al., 2016; Vaz et al., 2016). The function of SPRTN outside of S phase is not yet characterized. SPRTN expression peaks during S and G2 phases of the cell cycle and targeted for degradation following mitosis by APC-Cdh1 (Mosbech et al., 2012). Synchronized SPRTN knockdown cells accumulate more DPCs during S phase and are only hypersensitive to DPC-inducing agents when they are in replicative cell cycle stages (Vaz et al., 2016). Importantly, SPRTN associates with the replication machinery. SPRTN immunoprecipitated with replication fork proteins, namely PCNA, MCM2, MCM6, and Polδ, and iPOND experiments revealed that SPRTN is present on nascent DNA (Vaz et al., 2016; Maskey et al., 2017; Halder et al., 2019). SPRTN depletion impairs replication progression, and treatment of cells with formaldehyde or CPT exacerbated the effects of SPRTN depletion on replication fork progression, and overexpression of SPRTN FL but not E112A rescued the defects, indicating that SPRTN travels with the replisome to degrade endogenous DPCs encountered by the fork (Vaz et al., 2016; Mórocz et al., 2017).

## SPRTN Mutation Causes Ruijs-Aalfs Syndrome

In 2014, Lessel et al. identified three patients from two families who presented with symptoms of an atypical Werner syndrome characterized by genome instability, early-onset hepatocellular carcinoma and segmental progeria (Lessel et al., 2014). Genome-wide linkage analysis and exome sequencing of the affected boy (A-IV:1) from Family A and one affected boy (B-II:4) from Family B revealed *SPRTN* as the only gene with rare biallelic mutations in the exomes of both individuals. A-IV:1 had a 1 bp deletion (c.721delA), which predicted a premature stop codon at aa 249. B-II:4 was compound heterozygous for two mutations: missense (c.350A > G) resulting in aa substitution Y117C, and a 4 bp deletion (c.717_718+2delAGGT) resulting in intron inclusion, producing a premature stop codon at aa 246 (Figure 4). The syndrome was called Ruijs-Aalfs syndrome (RJALS), named for the authors of the clinical report characterizing the first identified patient (Ruijs et al., 2003). Primary skin fibroblasts from RJALS patients displayed reduced cell proliferation. Lymphoblastoid cells from patients showed increased percentages of stalled replication forks, new origin firing, chromosome instability and displayed a G2/M checkpoint defect, enhanced upon treatment with the DNA damaging agents. The high levels of replication stress in RJALS patient cells were linked directly to SPRTN mis-regulation. Silencing of the SPRTN ortholog in zebrafish (*sprtn*) resulted in an increase of γH2AX foci, early mortality, and delayed development, which was rescued with complementation of SPRTN full length (FL), but not Y117C or ΔC RJALS SPRTN mutants (Lessel et al., 2014). *Sprtn* hypomorphic mice had reduced size, and by 12 months of age displayed a progeroid phenotype: lordokyphosis, cataracts, reduced fat mass, reduced exercise ability, and increased levels of senescence markers. Lung fibroblasts from *Sprtn* hypomorphic mice displayed defects in replication fork progression, chromosomal instabilities, aneuploidy (Maskey et al., 2014). Liver tissues from 22 to 25-month-old *Sprtn* hypomorphic mice had increased levels of aneuploidy and the mice had an increased incidence of spontaneous tumor formation, many of which formed in the liver (Maskey et al., 2017). Thus, SPRTN mutation result in genome instability, early-onset hepatocellular carcinoma and progeria underscoring the importance of both N-terminal SprT domain and C-terminal protein-protein interaction domains of SPRTN in SPRTN function.

## Regulation of SPRTN Protease and SPRTN-Mediated DPC Proteolysis

SPRTN protease activity must be tightly regulated to prevent aberrant proteolysis of functional DNA-binding proteins. The sequence non-specificity of SPRTN protease and the lack of a distinct binding pocket at the catalytic active site indicates that regulation must come from other sources, such as protein-protein interaction, DNA binding, and post-translational modifications that modulate the ability of SPRTN to localize to DPC damage sites, accurately recognize and cleave substrates, or undergo auto-cleavage.

### SPRTN Function is Dependent on C-Terminal Domains

RJALS SPRTN Y117C mutant protein can form CPT-induced damage foci, but SPRTN ΔC mutant protein does not (Lessel et al., 2014). SPRTN ΔC is mislocalized to the cytoplasm, presumably due to the loss of the nuclear localization signal (NLS). Mutation of the NLS in SPRTN FL protein resulted in mislocalization to the cytoplasm, and the cells were unable to repair DPCs (Lopez-Mosqueda et al., 2016). The addition of a NLS to SPRTN ΔC restored its localization the nucleus and restored Histone H3 cleavage in cells (Lopez-Mosqueda et al., 2016), likely due to the presence of the DNA binding regions. Although SPRTN ΔC does have protease activity (Stingele et al., 2016; Vaz et al., 2016), it can only partially rescue effects of DPC induction (Lopez-Mosqueda et al., 2016; Stingele et al., 2016; Vaz et al., 2016), suggesting that the C-terminus is required for faithful DPC repair.

The lack of a substrate cleavage consensus motif and the severe effects of the RJALS SPRTN ΔC mutant suggest that SPRTN recruitment and function at DPCs is regulated in part by protein-protein interactions dependent on the C-terminus of SPRTN (Table 1). SPRTN SH and PIP mutants were enriched at the chromatin upon formaldehyde treatment and proficient in DPC proteolysis, but mutation of the UBZ domain, while still enriched at the chromatin upon formaldehyde treatment, was unable to cleave DPCs (Stingele et al., 2016; Larsen et al., 2019). Ubiquitinated DPCs induced by formaldehyde treatment are recognized by the SPRTN UBZ motif and the UBZ domain is required for its proper localization to ubiquitinated DPC repair sites. However, overexpression of SPRTN ΔUBZ mutant in *SPRTN* depleted cells only partially rescued DPC levels (Ruggiano et al., 2021). Studies in *Xenopus* extracts revealed that proficient cleavage of DPCs by SPRTN occurs even when DPC ubiquitination is prevented (Larsen et al., 2019). These observations indicate that not all DPCs generated by formaldehyde treatment or non-specific crosslinkers are ubiquitinated. DPCs can remain unmodified or receive other posttranslational modifications which are recognized by DPC sensors. Thus, in addition to the UBZ domain which is critical for SPRTN recruitment to ubiquitinated DPC sites, SPRTN can recognize or localize to DPCs by interacting with other DPC sensors via SPRTN C-terminal domain.

**TABLE 1:** Mechanism of SPRTN regulation. Summary of mechanisms of SPRTN regulation, including a summary statement and relevant SPRTN posttranslational modifications, protein-protein or protein-DNA interactions. See main text for expanded details. Key references are indicated in the right-most column.

Mechanism of SPRTN regulation	Summary	Key interactions	References
C-terminal protein interactions	C-terminal domains promote nuclear localization, replication fork association, damage localization, and DPC proteolysis	PCNA, VCP, TEX264, Ubiquitin	(Centore et al., 2012; Ghosal et al., 2012; Juhasz et al., 2012; Lessel et al., 2014; Lopez-Mosqueda et al., 2016; Stingele et al., 2016; Larsen et al., 2019; Fielden et al., 2020; Ruggiano et al., 2021)
DNA Binding	Two-factor DNA binding by the BR and ZBD regions of SPRTN activates protease activity	ss/dsDNA junctions and other atypical DNA structures	(Vaz et al., 2016; Toth et al., 2017; Reinking et al., 2020b)
Phosphorylation	CHK1-mediated SPRTN phosphorylation at S373, S374 and S383 promotes SPRTN chromatin association	CHK1	Halder et al. (2019)
Monoubiquitin switch	SPRTN monoubiquitination is a positive regulator of auto-cleavage activity. Deubiquitination of SPRTN upon DPC-induction reduces SPRTN auto-cleavage	USP11, USP7	(Perry et al., 2021; Zhao et al., 2021)
Acetylation	VCPIP1-mediated SPRTN deubiquitination promotes SPRTN acetylation and possible chromatin retention	VCPIP1, PCAF, GCN5	Huang et al. (2020)

In contrast to observations that interaction with VCP, a AAA-ATPase that utilizes ATP hydrolysis to extract ubiquitinated proteins from cellular structures is dispensable for SPRTN-mediated DPC repair, it was reported that SPRTN SH mutant cannot rescue TOP1-cc accumulation in SPRTN knockdown cells (Hänzelmann and Schindelin, 2017; Ye et al., 2017; Fielden et al., 2020). It was simultaneously shown that VCP is recruited to TOP1-ccs by the VCP cofactor TEX264 interacting with SUMO-1 modified TOP1-ccs. TOP1, VCP, and TEX264 were identified as SPRTN interacting partners, and the SPRTN-TOP1 interaction was decreased upon knockdown of TEX264. Interestingly, *in vitro* SPRTN proteolysis of TOP1-ccs was increased when TOP1-ccs were pre-incubated with TEX264 and VCP, indicating that TOP1-cc remodeling by VCP and its cofactor TEX264, in advance of cleavage by SPRTN, is required for efficient TOP1-cc repair. TEX264 appears to be specific to TOP1-ccs, as knockdown of TEX264 did not induce global accumulation of DPCs as knockdown of SPRTN does (Fielden et al., 2020). SPRTN has also been shown to interact with VCP cofactors UFD1-NPL4 (Davis et al., 2012), but further studies are needed to investigate whether SPRTN-mediated cleavage of other DPCs is assisted by VCP remodeling.

### Two-Factor, Structure-Specific DNA Binding Activates SPRTN Protease Activity

SPRTN is a DNA-dependent metalloprotease (Lopez-Mosqueda et al., 2016; Vaz et al., 2016). Mutational analysis revealed that SPRTN binds DNA via aa 200–250, and that these residues are required for SPRTN protease activity (Stingele et al., 2016; Vaz et al., 2016). This segment of SPRTN contains the basic region (BR), a predicted DNA binding region from aa 220–230 (Figure 3A) (Toth et al., 2017; Li et al., 2019). Mutation of the BR prevented SPRTN autocleavage, conferred formaldehyde sensitivity to cells, impaired formaldehyde-induced DPC repair, and reduced SPRTN in the chromatin fraction (Mórocz et al., 2017; Toth et al., 2017). DNA binding by the BR is assisted by multiple other regions of SPRTN. Mutations within the ZBD disrupted DNA binding, auto-cleavage, and Histone H1 cleavage in the absence of the BR (Li et al., 2019). SPRTN ΔC had reduced DNA binding (Lopez-Mosqueda et al., 2016; Vaz et al., 2016; Toth et al., 2017), indicating that the C-terminus harbors some accessory DNA binding function (Li et al., 2019). DNA binding activates SPRTN protease activity by acting as a scaffold that brings SPRTN and its substrate into proximity (Vaz et al., 2016), and induces a conformational change in SPRTN promoting flexibility and a more open conformation of SPRTN (Stingele et al., 2016; Vaz et al., 2016; Li et al., 2019).

*In vitro* assays investigating SPRTN substrate cleavage and auto-cleavage have shown a variety of results regarding DNA structure specificity for SPRTN protease function. There are reports that substrate cleavage occurs primarily in the presence of ssDNA (Stingele et al., 2016; Mórocz et al., 2017), while others show effective substrate cleavage using dsDNA in the reactions (Lopez-Mosqueda et al., 2016; Vaz et al., 2016; Halder et al., 2019). To delineate a more precise picture of DNA activation of SPRTN, Reinking *et al.* performed detailed *in vitro* cleavage assays with highly specific DNA structures (Reinking et al., 2020b). SPRTN auto-cleavage was active in the presence of dsDNA ends. Long stretches of dsDNA decreased SPRTN auto-cleavage, and SPRTN cleaved substrates bound to both the 5ʹ and 3ʹ ends of dsDNA, but it was not activated if the protein was crosslinked within a stretch of unperturbed dsDNA. SPRTN substrate cleavage was robustly active when the protein was crosslinked in close proximity to ss/dsDNA junctions and hairpins. SPRTN cleaved substrates bound across from DNA nicks, gaps, bubbles, and recessed ends up to 5 bp away, suggesting that the presence of atypical DNA structure is a major factor contributing to SPRTN substrate specificity (Reinking et al., 2020b).

Mutations in the ZBD or the BR of SPRTN reduced auto-cleavage or substrate cleavage *in vitro*. In cells, the mutants also displayed reduced auto-cleavage, but their accumulation at the chromatin upon formaldehyde exposure was unaffected, suggesting that SPRTN is initially recruited by protein-protein interactions. NMR analysis of the DNA binding capacity of SPRTN ZBD or BR mutants revealed transient and dynamic contacts of DNA with the ZBD and BR. The BR primarily bound to the backbone of dsDNA, and the ZBD likely binds to unpaired bases at the ends of dsDNA such as frayed ends, ss/dsDNA junctions, and ends of hairpins (Reinking et al., 2020b).

Collectively, these data support a model where SPRTN is recruited to damage sites by protein-protein interactions mediated by the C-terminal domains, followed by DNA binding by two regions of SPRTN: the ZBD and BR (Figure 3A and Table 1). This dual-factor DNA interaction for protease activation may ensure that the appropriate DNA structure is present at the cleavage site, serving as a protective measure against aberrant cleavage of functional DNA binding proteins. In agreement, SPRTN-mediated DPC degradation, but not proteasome-mediated, was shown to require nascent strand extension to within a few nucleotides of the DPC, and inhibition of gap filling across from a ssDNA-bound DPC prevented SPRTN-mediated DPC degradation (Larsen et al., 2019). The ss/dsDNA junction formed by the nascent DNA synthesis may be the appropriate activator of SPRTN protease activity.

### An SPRTN-CHK1 Cross-Activation Loop Regulates SPRTN Function

The ATR-CHK1 signaling cascade is a regulator of the replication stress response, where it regulates origin firing, fork stability, and delays mitotic entry by preventing CDK1/2 hyperactivation (Zeman and Cimprich, 2014; Zhang and Hunter, 2014). ATR-CHK1 kinase activity is required for physiological DNA replication but the whole cascade is not robustly activated, and there are open questions as to how CHK1 is activated and evicted from replicating chromatin without significant upstream signaling of the canonical ATR-CHK1 effector proteins (Petermann et al., 2006; Smits et al., 2006).

SPRTN depletion caused shortened DNA fiber track lengths, an increased percentage of stalled replication forks, and accumulation of chromosomal aberrations (Halder et al., 2019). Interestingly, new origin firing was robustly increased rather than suppressed, indicative of defective ATR-CHK1 signaling (Syljuåsen et al., 2005). Indeed, despite showing signs of replication stress, SPRTN-depleted cells did not activate CHK1, and downstream targets such as CDC25A degradation and inactivation of CDK1/2 were stabilized and hyperactive, respectively (Halder et al., 2019). CHK1 accumulated on both the nascent and mature chromatin in SPRTN-depleted cells during S-phase, which was rescued by ectopic expression of SPRTN FL but not E112A. *In vitro* and cellular experiments showed that SPRTN cleaves CHK1 in its C-terminus, leaving the N-terminal kinase region intact and more active than CHK1 FL. Expression of N-terminal CHK1 cleavage products restored fork velocity and suppressed new origin firing and fork stalling in SPRTN-depleted cells (Halder et al., 2019).

In addition to SPRTN promoting CHK1 cleavage and chromatin eviction, expression of CHK1 FL or N-terminal cleavage products promoted SPRTN chromatin association to a similar degree as formaldehyde treatment. Mass spectrometry analysis of SPRTN phospho-peptides from cells expressing FL or kinase-defective CHK1 identified 3 putative SPRTN phosphosites: S373, S374, and S383 (Figure 3A). Mutation of any of the SPRTN phosphosites or treatment with CHK1 inhibitor UCN-01 reduced SPRTN phosphorylation. Expression of phospho-mimetic, but not phospho-mutant SPRTN constructs rescued the replication defects observed in SPRTN-depleted cells and reduced CHK1 chromatin accumulation, suggesting the presence of an SPRTN-CHK1 cross-activation loop (Halder et al., 2019). Collectively, these results indicate that SPRTN protease activity is required to maintain steady-state replication, and that phosphorylation of SPRTN by CHK1 strengthens its association with the chromatin (Table 1).

### SPRTN Auto-Proteolysis is Regulated by a Monoubiquitin Switch in DPC Repair

Perhaps the most interesting mechanism of SPRTN regulation is its dynamic post-translational modification by monoubiquitin. Two early proteomic studies prior to SPRTN characterization identified C1orf124 ubiquitination (Emanuele et al., 2011; Kim et al., 2011), which was confirmed in several of the initial reports on SPRTN function in DNA damage tolerance (Centore et al., 2012; Mosbech et al., 2012). Mass spectrometry experiments revealed four potential SPRTN ubiquitination sites: K341, K376, K414, and K435. However, mutation of these four residues did not abolish SPRTN monoubiquitination. The C-terminus of SPRTN is lysine-rich, and mutation of 10 C-terminal lysine residues within SPRTN did not abolish SPRTN monoubiquitination but did result in a largely unstable protein (Stingele et al., 2016).

Interestingly, SPRTN is monoubiquitinated in a UBZ domain-dependent manner, as mutation of two cysteine residues to alanine in the UBZ domain resulted in a loss of SPRTN monoubiquitination (Centore et al., 2012; Mosbech et al., 2012). UBZ domain-dependent monoubiquitination has been proposed as an autoinhibitory function for various proteins, whereby intramolecular interaction between a UBZ domain and a conjugated ubiquitin prevents UBZ domain interaction with ubiquitinated proteins *in trans* (Hoeller et al., 2006; Bienko et al., 2010). The promiscuity of the SPRTN monoubiquitination site makes intramolecular UBZ-ubiquitin binding an attractive hypothesis, as it suggests that SPRTN monoubiquitination could be less of a direct activation signal and more of a physical barrier acting independently of precise protein conformation.

A ubiquitin-fused SPRTN construct was generated to investigate whether the UBZ domain of SPRTN can recognize its own monoubiquitin. Ub-fused SPRTN did not form foci in response to UV damage, was not monoubiquitinated, and displayed a defect in ubiquitin binding. Conversely, when SPRTN was fused to ubiquitin harboring an I44A mutation that prevents interaction with UBZ domains (Bomar et al., 2007), UV-induced foci formation, monoubiquitination, and ubiquitin binding functions were restored (Mosbech et al., 2012), suggesting that SPRTN monoubiquitination binds the UBZ domain and shields it from interacting with ubiquitinated proteins at UV damage sites.

Although a large fraction of the SPRTN cellular pool is not ubiquitinated under steady-state conditions, it would be reasonable to hypothesize that if monoubiquitinated SPRTN is unable to localize to UV damage sites, then widespread induction of UV damage would result in deubiquitination of SPRTN. However, UV exposure did not induce detectable levels of SPRTN deubiquitination in cells, but unmodified SPRTN was enriched at the chromatin upon UV damage (Stingele et al., 2016). In the context of DPC induction, SPRTN deubiquitination was observed upon treatment with formaldehyde and high doses of CPT and cisplatin (Stingele et al., 2016; Huang et al., 2020). Unmodified SPRTN was enriched at the chromatin upon formaldehyde treatment, suggesting the presence of a DPC-specific ubiquitin switch, where DPC induction triggers deubiquitination of SPRTN and subsequent recruitment to the chromatin (Stingele et al., 2016).

Several mass spectrometry analyses and DUB screens searching for novel SPRTN interactors identified the deubiquitinases VCPIP1, USP7, and USP11 as SPRTN-interacting proteins (Ghosal et al., 2012; Huang et al., 2020; Perry et al., 2021; Zhao et al., 2021). These three DUBs were each shown to deubiquitinate SPRTN in cells and *in vitro*, and loss of one or more of the DUBs prevented SPRTN deubiquitination upon treatment with DPC-inducing agents (Huang et al., 2020; Perry et al., 2021; Zhao et al., 2021). Huang *et al.* reported that knockdown of VCPIP1 prevented SPRTN chromatin localization upon formaldehyde treatment, in agreement with previous reports that monoubiquitinated SPRTN is excluded from the chromatin (Stingele et al., 2016; Huang et al., 2020). In contrast, Zhao *et al.* and Perry *et al.* showed that in the absence of USP7, USP11, or VCPIP1, both unmodified and monoubiquitinated SPRTN was enriched at the chromatin, suggesting that deubiquitination of SPRTN is not required for its chromatin association (Perry et al., 2021; Zhao et al., 2021). Instead, monoubiquitination was implicated as a regulator of SPRTN stability. Cycloheximide chase experiments showed that monoubiquitinated SPRTN had a shorter half-life than unmodified SPRTN, and SPRTN levels were stabilized by MG132 treatment. Thus, monoubiquitin may prime SPRTN for polyubiquitination and subsequent proteasomal degradation (Zhao et al., 2021). Additionally, deubiquitination of SPRTN was shown to be a negative regulator of SPRTN auto-cleavage activity, as overexpression of USP7 or USP11, but not catalytic inactive point mutants, reduced SPRTN auto-cleavage. USP11 overexpression has a more dramatic effect on SPRTN auto-cleavage suppression than USP7 does (Perry et al., 2021). Knockdown of the identified DUBs for SPRTN increased the levels of SPRTN auto-cleavage (Perry et al., 2021; Zhao et al., 2021). Inhibition of E1 ubiquitin activating enzymes, which abolished SPRTN monoubiquitination, also prevented SPRTN auto-cleavage (Zhao et al., 2021). These results support an updated model of SPRTN regulation by deubiquitination during DPC repair, where DPC induction triggers SPRTN deubiquitination, which increases the affinity of SPRTN protease activity towards DPCs rather than itself. In the absence of DUB activity, SPRTN auto-proteolysis is high, reducing the pool of SPRTN that is available for DPC proteolysis, leading to delayed DPC repair, genomic instability, and cell death (Table 1).

It is unclear whether the three identified SPRTN DUBs act redundantly or in isolation. It is likely that in the case of loss of one or more DUBs, the remaining DUB(s) can compensate to some degree. However, considering that a functional deficit is observed upon single knockdown of any of the three DUBs upon formaldehyde treatment as well as DPC accumulation and cell death after treatment with multiple DPC-inducing agents in USP11 or USP7 single knockdown cells, there appears to be some level of non-redundancy for each DUB.

To date, no E3 ubiquitin ligase specific to SPRTN has been identified and attempts to map the ubiquitination site have revealed that SPRTN monoubiquitination is promiscuous, as mutation of multiple residues in the lysine-rich C-terminus of SPRTN results in protein destabilization before it abolishes monoubiquitination (Stingele et al., 2016; Zhao et al., 2021). Interestingly, it has been shown that some proteins harboring ubiquitin binding domains may undergo E3-independent monoubiquitination, where UBDs interact with ubiquitin-primed E2 conjugating enzymes to mediate auto-ubiquitination *in cis* (Hoeller et al., 2007). An *in vitro* panel identified ten recombinant E2 ligases that could induce SPRTN E112Q monoubiquitination in the absence of an E3 ubiquitin ligase (Zhao et al., 2021). However, these results were not investigated in cells; thus, the possibility of E3 dependency for SPRTN monoubiquitination *in vivo* cannot be excluded.

### SPRTN Recruitment to Chromatin

SPRTN associates with the replisome as it interacts with a variety of replication fork proteins and is present on nascent DNA, even in the absence of damage (Vaz et al., 2016; Maskey et al., 2017). Both monoubiquitinated and unmodified SPRTN are localized on chromatin in the absence of damage and SPRTN phosphorylation by CHK1 stimulated SPRTN chromatin recruitment to promote unperturbed DNA replication fork progression and DPC repair (Halder et al., 2019). Huang *et al.* showed that in addition to SPRTN deubiquitination, VCPIP1 promoted SPRTN interaction with the acetyltransferases PCAF/GCN5. Antibodies specific to acetylated residues revealed SPRTN acetylation upon formaldehyde treatment, and mass spectrometry analysis identified K230 as an acetylation site (Huang et al., 2020). This conserved residue is located within the BR of SPRTN, a critical DNA binding motif (Toth et al., 2017; Li et al., 2019). Mutation of the SPRTN acetylation site (SPRTN K230R) prevented SPRTN chromatin accumulation upon formaldehyde treatment, and SPRTN K230R expression did not repair formaldehyde-induced DPCs or rescue formaldehyde or CPT cellular sensitivity. Knockdown of PCAF and GCN5 resulted in reduced SPRTN acetylation and chromatin association but not formaldehyde-induced deubiquitination, suggesting that SPRTN acetylation is secondary to deubiquitination (Huang et al., 2020). Thus, the identification of VCPIP1 as a dual promoter of SPRTN deubiquitination and acetylation may mean that knockdown of VCPIP1 is dysregulating SPRTN at multiple steps, and that SPRTN acetylation may be the signal for its chromatin retention upon DPC induction rather than deubiquitination (Table 1). Thus, SPRTN deubiquitination and chromatin enrichment upon DPC induction are independent processes. Phosphorylation, but not deubiquitination of SPRTN could promote SPRTN recruitment to chromatin in undamaged conditions and acetylation may retain SPRTN at the chromatin when cells are burdened with high levels of DPCs (Halder et al., 2019; Huang et al., 2020). It was also shown that SPRTN is recruited via the UBZ domain to ubiquitinated DPCs for DPC removal, while SUMOylation of DPCs suppresses repair by HR and promotes DPC repair by SPRTN (Ruggiano et al., 2021). The precise mechanism underlying SPRTN recruitment to DPC lesions is still largely unclear. The crosstalk between the posttranslational modifications such as phosphorylation and acetylation of SPRTN and ubiquitination and SUMOylation of DPCs mediating the recruitment of SPRTN to DPC sites is still not known.

## Replication-Independent DPC Removal

Among the different types of DPCs, repair of TOP-ccs has been extensively investigated. Genetic and biochemical experiments performed in bacteria, yeast, mammalian cell lines, and with recombinant proteins *in vitro* indicate that NER and HR pathway can repair DPCs (Chanet et al., 1976; Minko et al., 2002; Minko et al., 2005; Reardon and Sancar, 2006; Baker et al., 2007; Nakano et al., 2007; Nakano et al., 2009; Scharer, 2013). In addition to utilizing components of canonical DNA repair pathways, tyrosyl-DNA phosphodiesterase 1 and 2 (TDP1 and TDP2) have been shown to mediate TOP1-cc and TOP2-cc repair, respectively (Yang et al., 1996; Pouliot et al., 1999; Ledesma et al., 2009; Zeng et al., 2011). We refer the readers to excellent review articles that describe replication-independent DPC repair by NER, HR, MRE11, TDP1 and TDP2 in detail (Sun et al., 2020a; Sun et al., 2020b; Wei et al., 2021; Weickert and Stingele, 2022). In this section we will discuss proteolysis of DPCs outside of S phase.

Early studies showed that the proteasome has a role in DPC proteolysis. Proteolytic digestion of a large DPC to a small peptide-DNA adduct promoted NER-mediated excision of the lesion (Baker et al., 2007), indicating that NER repair factors are likely unable to access the DNA because of steric hindrance with larger DPCs. Similarly, TOP1 or TOP2 protein must be degraded by the proteasome in order for TDP1 or TDP2-mediated hydrolysis of the buried covalent crosslink. CPT treatment of human cell lines induced downregulation of TOP1 protein levels, which was inhibited by treatment with MG132. MG132 treatment also resulted in accumulation of polyubiquitinated TOP1 upon CPT treatment, indicating that the 26S proteasome degrades TOP1-ccs (Desai et al., 2001). Similarly, TOP2 is degraded in response to VP16 treatment, dependent on the activity of the 26S proteasome (Mao et al., 2001).

One potential DPC protease has been investigated for replication-independent DPC repair. GCNA (germ cell nuclear acidic peptidase) is a conserved regulator of genome stability across eukaryotic organisms. Human GCNA is an SprT domain-containing putative protease, and in lower eukaryotes, *Gcna* mutants accumulated DPCs (Carmell et al., 2016; Bhargava et al., 2020; Dokshin et al., 2020). Human GCNA may be a tumor suppressor, as GCNA had a high alteration frequency in an analysis of human germ cell tumors (Bhargava et al., 2020). Ectopically expressed GCNA colocalized with SUMO in response to formaldehyde treatment and colocalized with DNMT1 in response to treatment with 5-aza-2′-deoxycytidine, a DNMT1 DPC inducing agent. These localizations were abolished by inhibition of SUMO but not ubiquitin. GCNA has multiple SUMO-interacting motifs (SIM). Mutation of these motifs abolished interaction with SUMO (Borgermann et al., 2019). However, detectable levels of endogenous GCNA were not present in human somatic cell lines, and ectopic expression of GCNA had a negative impact on DNMT1 repair and cellular survival, indicating that GCNA function in human cells is tightly restricted to the germline (Carmell et al., 2016; Borgermann et al., 2019). Thus, replication-independent DPC degradation in human somatic cells has been, to date, only characterized to occur via the proteasome.

In addition to early evidence that the proteasome contributes to DPC degradation, post-translational modification of TOP1-ccs and TOP2-ccs by SUMO in human cells has been observed to regulate DPC degradation outside of DNA replication (Mao et al., 2000; Mo et al., 2002). Formaldehyde treatment of human cells caused an accumulation of SUMO1 and SUMO2/3-modified chromatin-bound proteins, independent of S-phase. A similar SUMO response was observed upon 5-aza-2ʹ-deoxycytidine treatment. The majority of SUMO-modified DNMT1 DPCs were present on mature chromatin, indicating they are modified independently of DNA replication. Interestingly, DNMT1 was ubiquitinated in a SUMO-dependent manner, and DNMT1 DPC removal was prevented with proteasome inhibition (Borgermann et al., 2019). Recent follow up studies showed that independent of replication, DNMT1 DPCs, TOP1-ccs, and TOP2-ccs are SUMOylated via the PIAS4 SUMO ligase and subsequently ubiquitinated by the SUMO-targeted E3 ubiquitin ligase RNF4 and targeted for proteasomal degradation (Sun et al., 2020c; Liu et al., 2021) (Figure 2A). SUMOylation has also been implicated in TOP2-cc repair independent of the proteasome. In the absence of proteasome activity, the SUMO ligase ZNF451 was shown to promote TOP2-cc SUMOylation that facilitated TDP2 activity independent of TOP2 degradation, although this is likely a secondary mechanism of TOP2-cc removal (Schellenberg et al., 2017).

## DNA-Peptide Adduct Removal: A Secondary Role of SPRTN in DPC Repair

Studies in *Xenopus* egg extracts showed that when the replisome collides with DPCs, the CMG helicase stalls and the DPC is proteolyzed into a peptide-DNA adduct that is bypassed by TLS polymerases (Duxin et al., 2014). In agreement, *in vitro* studies with human TLS polymerases suggested that DPCs must be proteolytically degraded to a small peptide adduct prior to bypass by TLS polymerases (Yeo et al., 2014). Notably, SPRTN depletion delayed polymerase extension beyond the lesion even when the proteasome was actively degrading DPCs, suggesting a two-factor role for SPRTN in DPC repair during DNA replication: DPC proteolysis and promotion of TLS (Larsen et al., 2019).

During TLS, replicative DNA polymerases get switched for TLS polymerases, which include the Y-family polymerases (Polη, Polι, Polκ, and REV1) and the B-family polymerase Polζ (Sale et al., 2012; Bertolin et al., 2015). The polymerase switch is promoted primarily by monoubiquitination of PCNA, the trimeric DNA sliding clamp essential for DNA replication, at K164 by the E3-ubiquitin ligase activity of RAD18 (Hoege et al., 2002; Stelter and Ulrich, 2003; Kannouche et al., 2004). SPRTN interacts with unmodified and ubiquitinated PCNA in cells (Ghosal et al., 2012; Juhasz et al., 2012), which is strengthened upon DNA damage (Mosbech et al., 2012). Structural analysis of the SPRTN PIP box in complex with PCNA revealed that Y331 of the SPRTN PIP box has a pivotal PCNA binding role, as it forms an intramolecular hydrogen bond with SPRTN N326. L328 and F332 of the SPRTN PIP box “plug in” to the hydrophobic binding pocket of PCNA (L47, L126, P129, P234, Y250), though with lower binding affinity than TLS polymerases Polη or Polι (Wang et al., 2016). Mutation of the SPRTN PIP box reduced interaction with unmodified PCNA, whereas mutation of the UBZ domain abolished interaction with ubiquitinated PCNA, and a PIP/UBZ double mutant reduced interaction with both forms of PCNA (Centore et al., 2012; Ghosal et al., 2012; Juhasz et al., 2012). In addition to interacting with ubiquitinated PCNA, the UBZ domain of SPRTN mediates its interaction with both K48 and K63 ubiquitin chains (Centore et al., 2012; Davis et al., 2012; Mosbech et al., 2012).

Consistent with an essential role in TLS, SPRTN knockdown cells displayed increased sister chromatid exchanges and increased UV-induced mutagenesis (Davis et al., 2012; Juhasz et al., 2012; Machida et al., 2012; Mosbech et al., 2012; Kim et al., 2013). However, the exact mechanism of SPRTN function in TLS is unclear. Interestingly, SPRTN displayed preferential interaction either for the replicative polymerase subunit POLD3 or the TLS polymerase Polη in undamaged and UV-treated cells, respectively (Ghosal et al., 2012). Knockdown of SPRTN resulted in a reduction of UV-induced Polη foci (Centore et al., 2012; Toth et al., 2017), and overexpression of SPRTN caused an increase of Polη foci both in undamaged and UV damaged cells, indicating that SPRTN promotes the switch from replicative to TLS polymerase (Juhasz et al., 2012). SPRTN is recruited to UV damage sites in a manner dependent on PCNA interaction. Conversely, it was reported that Polη foci formation in response to UV and laser micro-irradiation damage foci was unaffected in SPRTN-depleted cells, but Polη retention at the damage site was instead prolonged (Davis et al., 2012; Mosbech et al., 2012; Maskey et al., 2014). Similar Polη damage site retention was observed when VCP was depleted, and VCP was defective in damage site localization in SPRTN knockdown cells (Davis et al., 2012; Mosbech et al., 2012). Prolonged UV-induced interaction between PCNA and Polη was reported in SPRTN knockdown cells, which was reduced with overexpression of SPRTN (Davis et al., 2012; Mosbech et al., 2012; Maskey et al., 2014), indicating SPRTN may recruit VCP to sites of polymerase switching to promote polymerase extraction from the chromatin. However, VCP has since been proposed to operate upstream of SPRTN for other types of DNA damage (Fielden et al., 2020). Although these observations were made in response to UV damage, similar effects cannot be ruled out when cells are exposed to DPC inducing agents. In agreement, a recent study showed that SPRTN is recruited to PARP1-DNA complex in S phase and catalyzes PARP1 proteolysis and replication bypass of PARP1-DNA complexes (Saha et al., 2021). Further work is required to elucidate the mechanistic function of SPRTN in TLS and replication bypass of different types of DPCs.

In addition to TLS, studies have proposed a role for HR in the removal of DNA-peptide adduct and/or repair of subsequent DNA breaks generated during DPC removal. Yeast strains deficient for NER displayed delayed removal of the bulk of DPCs induced by formaldehyde exposure which correlated with delayed entry into S-phase. Conversely, strains deficient for HR displayed G2 arrest upon formaldehyde exposure, indicating that the bulk of DPCs induced by formaldehyde are removed by NER outside of S-phase, but the lesions that are present during S-phase are dependent in part on HR (Stingele et al., 2014). Similarly, formaldehyde-induced DPC repair in mammalian cells is dependent on HR pathway (Nakano et al., 2009). No precise mechanistic role for canonical HR in DPC repair has been identified. However, certain HR components have been mechanistically characterized for their ability to remove DPCs. Specifically, the MRE11-RAD50-NBS1 (MRN) complex has been shown to remove TOP2-ccs in multiple organisms (Woodworth and Kreuzer, 1996; Keeney et al., 1997; Stohr and Kreuzer, 2001; Connelly et al., 2003; Neale et al., 2005; Hartsuiker et al., 2009a; Hartsuiker et al., 2009b; Aparicio et al., 2016; Deshpande et al., 2016; Hoa et al., 2016). Whether MRN can remove DPCs other than TOP2-ccs is not well characterized. Depletion of MRE11 did not result in accumulation of global DPCs (Vaz et al., 2016), but *in vitro* activity of MRN on DPC substrates was not strictly dependent on protein identity (Sacho and Maizels, 2011; Deshpande et al., 2016), and loss of the yeast MRN complex conferred sensitivity to formaldehyde to a greater extent than loss of other HR repair factors (de Graaf et al., 2009), indicating that MRN may have a role in DPC repair outside of TOP2-ccs. Although MRE11 is a substrate of SPRTN, it is not known whether SPRTN and MRN complex are epistatic in DPC repair (Na et al., 2021).

TDP1 and TDP2 can also participate in the removal of DNA-peptide adducts generated following TOP-cc proteolysis by SPRTN. SPRTN and TDP1 double knockdown cells were similarly sensitive to CPT as single knockdown cells, indicating that following proteolysis of TOP1 by SPRTN TDP1 hydrolyzes the phosphotyrosyl bond in the crosslink releasing TOP1 peptide from TOP1-DNA peptide adduct (Vaz et al., 2016; Maskey et al., 2017).

## Conclusion

Advances made in the last decade have led to the identification of key DPC repair enzymes and elucidated some of the mechanistic details in the DPC repair pathway. However, the precise mechanism of DPC repair is still unknown. Here, we discuss some open questions regarding DPC repair and SPRTN regulation which should be the focus of future investigations.

Several different types of DPCs are generated in the cell by endogenous and exogenous DPC inducing agents. How are different types of DPCs recognized? Post-translational modification of DPCs by ubiquitin and SUMO has been shown to regulate pathway choice during DPC repair. Ubiquitination and SUMOylation posttranslational modification occurs in proteins irrespective of whether the proteins are crosslinked to DNA, raising several questions about the specificity of these processes for DPC repair. How do DPC sensors distinguish ubiquitinated or SUMOylated DPCs from similarly modified proteins in the vicinity of the DNA or DNA binding proteins? Do all DPCs undergo post-translational modification? What factors govern whether a subset of DPCs is ubiquitinated or SUMOylated? Are there DPC type specific sensors and effectors present that transduce the signal to SPRTN and/or the proteasome for DPC proteolysis? Given the different types of DPCs, future studies should include the identification of novel DPC proteases.

Why do multiple DUBs regulate SPRTN? The identification of multiple SPRTN DUBs reveals a complex landscape of SPRTN regulation in steady-state conditions as well as in DPC repair. Foremost, the ability of at least three DUBs to deubiquitinate SPRTN highlights how critical the monoubiquitin switch and suppression of SPRTN auto-cleavage in damage conditions is for genomic stability. In contrast, it is likely equally critical that robust auto-cleavage is active in undamaged conditions, as indicated by the substantial fraction of SPRTN that is constitutively monoubiquitinated in undamaged cells. Basal levels of SPRTN auto-cleavage may prevent aberrant proteolytic cleavage of functional DNA-binding proteins or components of the replisome. SPRTN repairs different types of DPCs. Therefore, SPRTN DUB selection may be context dependent and future studies should investigate the signals that activate the different SPRTN DUBs in response to specific types of DPCs and whether they work cooperatively or in isolation to regulate SPRTN activity.

What is the fate of SPRTN following DPC cleavage? The exact role of SPRTN in TLS following DPC proteolysis is unclear. SPRTN can interact with DNA polymerases, PCNA, and VCP, indicating that following DPC cleavage, DNA-bound SPRTN may serve a scaffolding role to promote VCP-mediated polymerase extraction and polymerase reloading. SPRTN deubiquitination at this stage could free its UBZ domain to bind monoubiquitinated PCNA. However, further studies are needed to clearly define a role for SPRTN in TLS after DPC cleavage.

SPRTN auto-proteolysis is presumed to be an auto-inhibitory function. Basal levels of SPRTN auto-cleavage is observed in undamaged conditions. Thus, it is possible that substrate cleavage by unmodified SPRTN during normal DNA replication triggers the pool of monoubiquitinated SPRTN to promote SPRTN auto-cleavage *in trans*. Deubiquitination of SPRTN upon DPC induction suppresses SPRTN auto-cleavage, which may be indicative of sustained SPRTN activity, as larger protein adducts may require several rounds of cleavage. This raises the interesting possibility that following auto-cleavage, protease-active SPRTN fragments may serve a functional purpose if they are able to properly localize either through DNA binding or SprT-domain mediated protein-protein interactions. These fragments could serve to perform peptide-DNA processing secondary to proteasome-mediated DPC cleavage. Alternatively, termination of TLS and helicase-polymerase recoupling may trigger SPRTN auto-proteolysis or proteasomal degradation to inactivate SPRTN after DPC repair, although the exact mechanisms of SPRTN inactivation remain to be fully elucidated.

How do regulatory mechanisms of SPRTN cooperate for effective DPC repair? Multiple post-translational modifications of SPRTN have been identified, including ubiquitination, phosphorylation, and acetylation that regulate SPRTN autoproteolysis, chromatin localization under unperturbed conditions and retention of SPRTN on chromatin upon DPC-induction, respectively. Considering the evidence suggesting that SPRTN acetylation occurs secondarily to DPC-induced SPRTN deubiquitination and promotes chromatin retention during DPC repair, it is possible that acetylation protects SPRTN from premature auto-cleavage and DNA dissociation, although the effects of acetylation on SPRTN DNA binding and auto-cleavage are unknown. It is possible that in addition to promoting UBZ-dependent protein-protein interaction, deubiquitination of SPRTN allows the freed lysine residue to undergo alternative post-translational modification, such as SUMOylation, methylation, or acetylation that would be prevented by the presence of ubiquitin. In addition, the crosstalk among the various known SPRTN modifications for effective DNA repair should be examined.

Future directions also include the search for additional SPRTN and DPC pathway regulators. This includes the characterization of SPRTN ubiquitin conjugation in cells. *In vitro* evidence indicates that SPRTN may be able to undergo auto-ubiquitination (Zhao et al., 2021), although it is likely that *in vivo,* SPRTN ubiquitination is carried out by an unidentified E3 ubiquitin ligase. Further, additional VCP cofactors or independent substrate recognition proteins that cooperate with SPRTN to promote DPC recognition and cleavage remain to be identified.

The precise mechanism of how DNA-peptide adducts generated following DPC proteolysis are processed, and how the resulting DNA breaks are repaired needs to be delineated. Identifying DPC repair sensors, effectors, and DPC repair enzymes and elucidating the molecular mechanism of DPC repair pathway will provide further insight on how cells maintain genome stability upon DPC induction and how this repair pathway could be targeted to sensitize tumor cells to DPC-inducing chemotherapies.
